# Statistical Modeling of SAR Images: A Survey

**DOI:** 10.3390/s100100775

**Published:** 2010-01-21

**Authors:** Gui Gao

**Affiliations:** National University of Defence Technology, Changsha 410073, China; E-Mail: dellar@126.com; Tel.: +86-0731-84576350; Fax: +86-0731-84573435

**Keywords:** synthetic aperture radar (SAR) images, statistical models, parameter estimation, probability density function (PDF), the product model

## Abstract

Statistical modeling is essential to SAR (Synthetic Aperture Radar) image interpretation. It aims to describe SAR images through statistical methods and reveal the characteristics of these images. Moreover, statistical modeling can provide a technical support for a comprehensive understanding of terrain scattering mechanism, which helps to develop algorithms for effective image interpretation and creditable image simulation. Numerous statistical models have been developed to describe SAR image data, and the purpose of this paper is to categorize and evaluate these models. We first summarize the development history and the current researching state of statistical modeling, then different SAR image models developed from the product model are mainly discussed in detail. Relevant issues are also discussed. Several promising directions for future research are concluded at last.

## 1. Introduction

Statistical modeling of SAR images is one of the basic problems of SAR image interpretation. It involves several fields such as pattern recognition, image processing, signal analysis, probability theory, and electromagnetic scattering characteristics analysis of targets *etc.* [1]. Generally speaking, statistical modeling of SAR images falls into the category of computer modeling and simulation. At present, one of the major strategies of SAR image interpretation is to use the methods of classical statistical pattern recognition, which are based on Bayesian Theory and can reach a theoretically optimal solution [1,2]. To utilize these methods for SAR image interpretation, a proper statistical distribution must be adopted to model SAR image data [1,2]. Therefore, in the past ten years, statistical modeling of SAR image has become an active research field [1].

Statistical modeling is of great value in SAR image applications. Firstly, it leads to an in-depth comprehension of terrain scattering mechanism. Secondly, it can guide the researches of speckle suppression [3–9], edge detection [10], segmentation [1,11–13], classification [14–17], target detection and recognition [14,18–20] for SAR images, *etc.* Finally, combining statistical model with ISAR target database can simulate various SAR images with variable parameters of aspect, terrain content, region position and SCR (signal to clutter ratio), so statistical modeling can provide numerous data for developing robust algorithms of SAR image interpretation [21].

The research on statistical modeling of SAR images may be traced back to the 1970s. With the acquisition of the first SAR image in the U.S., the analysis of real SAR data directly promoted the development of statistical modeling techniques. The speckle model of SAR images, proposed by Arsenault [22] in 1976, is the origin of these techniques, which established the theoretical foundation of the later researches. In 1981, Ward [23] presented the product model of SAR images, which took the speckle model as a special case. As a landmark of the development of statistical modeling, the product model simplified the analysis of modeling. Since then, many scholars joined this research field and many statistical models of SAR images had been developed.

Since the 1990s, with the coming forth of a series of air-borne or space-borne SAR platforms, the acquisition of SAR data is no longer a problem. Due to the urgent demands for analyzing and interpreting the obtained image data, statistical modeling has drawn much attention.

In recent years, many famous research organizations have been studying SAR statistical modeling [24], and great progress has been made. According to the collected literatures, from 1986 to 2004, there were more than 100 papers dealing with SAR statistical modeling published in some famous journals such as *IEEE-AES*, *IEEE-IP*, *IEEE-GRS*, and *IEE*, *etc.* and at some international conferences such as SPIE and IGARSS. The related papers, which use SAR statistical model for the purpose of segmentation, speckle suppression, classification and target detection and recognition, are uncountable. Much creative research has been made. Professor Oliver, an English scholar, published his monograph *Understanding Synthetic Aperture Radar Images* in 1998 [1]. The book includes 14 chapters, two of which discuss the statistical modeling technology. It summarizes related techniques on SAR statistical modeling before 1997. After 1997, papers on SAR statistical modeling have appeared in renowned journals almost every year. The most attractive achievement among them is the statistical modeling on extremely heterogeneous region of SAR images proposed by Frery [24], who works in Brazil and has introduced the original idea that for the purpose of statistical modeling, SAR images can be divided into homogeneous regions, heterogeneous regions and extremely heterogeneous regions, according to their contents. Furthermore, statistical modeling of SAR images is taken as one of the main contents in more than 20 doctoral dissertations found in UMI and in the research reports from the Belgian Royal Military Academy. While numerous statistical distributions have been proposed to model SAR image data, we are unaware of any surveys on this particular topic. It is necessary to categorize and evaluate these models and relevant issues. The main contribution of this survey is the classification and evaluation of the statistical models of SAR images existed currently. The vital and latest contributions have also been covered in this paper. The survey is organized as follows: Section 2 illustrates the classification and the research contents of statistical modeling. In Sections 3 and 4, current statistical models are discussed in detail. The relationship of them and their limitations in applications are pointed out in Section 5. Major conclusions and developing trends of statistical modeling are also presented by Section 6. We conclude the survey in the final section.

## 2. Model Classification and Research Contents

According to the modeling process, the statistical models of SAR images can be divided into two categories [2,25–28]: parametric models and nonparametric models. When dealing with a parametric model, several known probability distributions of SAR imagery are given at first. Usually, the parameters of these distributions are unknown and have to be estimated according to the real image data. Finally, by using some certain metrics, the optimal distribution is chosen as the statistical model of the image. While handling a nonparametric model, no distributions have to be assumed, and the optimal distribution is obtained in a way of data-driven of image data. The merit of the nonparametric models is that they make the process of statistical modeling more flexible and can fit the real data more accurate.

**Figure 1.**
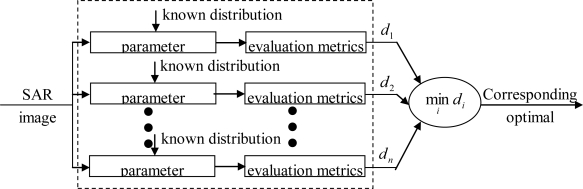
A general flow chart of parametric modeling.

Since nonparametric modeling involves complex computation as well as numerous data, it is usually time-consuming and cannot satisfy the requirements of various applications [25]. Consequently, parametric modeling is intensively studied. The process of parametric modeling can be described in brief as to choose an appropriate one from several given statistical distributions for the image to be modeled. The process is shown in Figure 1. According to Figure 1, the process of parametric modeling consists of: (1) analyzing several known statistical distribution models; (2) parameter estimation: estimating the parameters of different distribution; (3) goodness-of-fit tests: assessing the accuracy of the given models fitting to the real data.

### 2.1. Parameter Estimation

Several strategies have been proposed in the literature to deal with parameter estimation [26]. The two most frequently used methods are probably the “method of moments” (MoM) [1,17,29] and the maximum-likelihood (ML) methodology [19,27,30]. Recently, the method of log-cumulants (MoLC) is also included as a possible parameter estimation approach [3,17,31].

### 2.2. Goodness-of-Fit Tests

A number of methods for quantitatively assessing the validity of statistical models in light of sample data have been developed over the last hundred years. Many of these methods place the problem in a statistical hypothesis testing framework, pitting a null-hypothesis *H*_0_, an assertion that the data were not generated according to the model, against an alternative hypothesis *H*_1_, an assertion that they are not. The methods are then implemented by computing some statistic of the random observations that has a known distribution if *H*_0_ were true. Values of this quantity close to zero are interpreted as evidence that *H*_0_ should be rejected in favor of *H*_1_. The purpose of these methods is to seek the model that best describes observed data from a set of specified models, irrespective of whether any model is actually a good fit to the data [32].

In summary, the major rules for assessing the fitting accuracy includes the *χ*^2^ matching test [32,33], AIC (Akaike information criteria) rule [34], *K-S* (Kolmogorov-Smirnov) test [32,35,36], *K-L* distance measurement [37,38], D’Agostino-Pearson test [2,32,39], and Kuiper tests [31] *etc.* The research on parameter estimation as well as accuracy assessment is relatively mature and will not be discussed further in this paper. Relevant literature [2,31,32] can be consulted for more information.

## 3. Statistical Models

The purpose of statistical modeling of SAR images is to determine a statistical model for single-polarimetric images or multi-polarimetric images. The multi-polarimetric SAR images are a combination of four basic kinds of polarimetric images represented by the scattering matrix. For any one of the polarimetric images, its statistical characteristics are no different from those of a single-polarimetric image. The single-polarimetric statistical model can be extended to describe the multi-polarimetric images [40–43]. Therefore, studying the statistical models of single-polarimetric SAR images is of basic significance. This section mainly discusses this kind of models.

It is more than 30 years since the SAR statistical model has been first studied. Researchers have proposed various statistical models, among which the statistical model family based on the product model outperforms other models [2], so we would like to comprehensively summarize current statistical models using the product-model-based ones as a thread.

### 3.1. Nonparametric Models

The nonparametric models are an effective kind of models which can estimate the probability density function (PDF) of SAR image data based on the nonparametric method. The basic idea is to use the weighted sum of different kernel functions to obtain the estimation of the statistical distribution. Typical methods include: the Parzen window technique [27,44,45] the artificial neural networks (ANN) method [46,47], the support vector machine (SVM) method [48–50] *etc.* The characteristic of the nonparametric models is that it is a data-driven model and suitable for estimating the complex unknown PDF. Nonparametric modeling has high estimation accuracy, but it usually needs a large sample data set as well as complex operations and is a time-consuming task. Consequently, it’s seldom used in any applications, except several reports focus on the problem of ship target detection in SAR images with simple sea backgrounds [44].

### 3.2. Parametric Models

The underlying idea of parametric modeling is to use the parameter estimation method to determine the statistical model of SAR image data according to some known distributions. During the past 20 years, the parametric model has been widely and thoroughly studied. With the analysis of data from different sensors and the scattering mechanism of different kinds of terrain, many concrete SAR statistical distributions for different cases have been proposed.

## 4. Classification of Parametric Models

**Figure 2.**
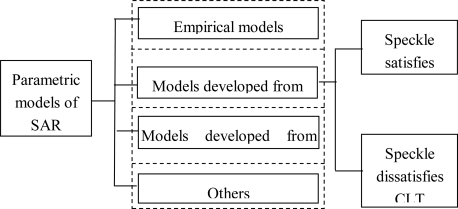
Four major categories of parametric modeling Note: PM represents the product model; CLT represents the central limit theorem; GCLT represents the general central limit theorem.

The parametric models can be classified into four categories according to its main idea (see Figure 2): (1) the empirical distributions; (2) the models developed from the product model (PM); (3) the models developed from the generalized central limit theorem (GCLT); 4) other models.

### 4.1. The Statistical Models Developed from the Product Model

**Figure 3.**
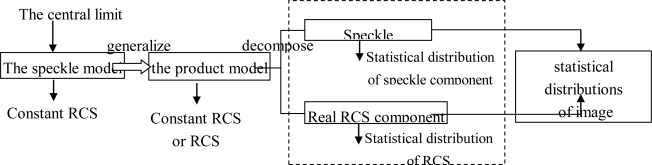
Process of developing a statistical model from the product model.

The product model is widely used in SAR image analyzing, processing and modeling. Most of the widely-used statistical models are developed from the product model, which is derived in turn from the speckle model. The process of developing concrete statistical models from the speckle model is shown in Figure 3.

The speckle model, proposed by Arsenault [22], is deduced from the coherent imaging mechanism of a SAR system, under the ideal circumstance that the imaged scene has a constant RCS (*i.e.*, speckle is fully developed and homogeneous surfaces appear as stationary fields).The deducing process based on the coherent imaging mechanism begins with the six reasonable hypotheses as follows [1,26,51,52]:

- Each resolution cell contains sufficient scatterers;
- The echoes of these scatterers are independently identically distributed;
- The amplitude and phase of the echo of each scatterer are statistically independent random variables;
- The phase of the echo of each scatterer is uniformly distributed in [0,2π];
- Inside a resolution cell, there are no dominant scatter- ers;
- The size of a resolution cell is large enough, compared with the size of a scatterer.

Secondly, with the six hypotheses mentioned above and the central limit theorem (CLT) [53], it can be proven that the energy of each resolution cell has a negative exponential distribution with the mean value equal to the real RCS value of the resolution cell. Finally, according to the hypothesis of constant RCS background, each resolution cell can be considered as a stochastic process, with the ergodic property (*i.e.*, each resolution cell is statistically independent). Therefore, the whole image has a distribution identical to that of a single resolution cell.

Motivated by the speckle model, Ward [23] proposed the product model of SAR images. Figure 3 shows the process of developing a statistical model from the product model. According to Figure 3, the product model combines an underlying RCS component *σ* with an uncorrelated multiplicative speckle component *n*, so the observed intensity *I* in a SAR image can be expressed as the product [38,54–58]:

(1) I=σ·n

The speckle model is taken as the special example of the product model with a constant RCS (σ). Because the product model is correlated with the underlying terrain RCS (σ), it is usually applied to the intensity data (energy or the square of amplitude). That is, *I* in Equation (1) represents the observed value of the intensity. The product model simplifies the analysis of the statistical model of SAR images. So it is widely used to develop models which take the RCS fluctuations into consideration. where *P* (σ) represents the RCS component distribution and *P* (*I* | σ) is correlated with the distribution of speckle component.

Since the speckle component has a determinate statistical distribution, only the RCS fluctuation component need to be considered when developing the statistical models of SAR images (see Figure 3). According to the product model in Equation (1), the PDF of the observed intensity is given by:

(2) $$P(I)=\int_{0}^{\infty }P(\sigma )P(I|\sigma )d\sigma$$

**Figure 4.**
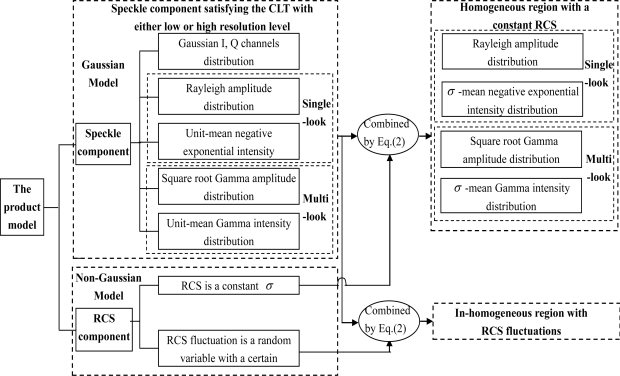
Statistical models of constant RCS or RCS fluctuations when the speckle component satisfy the central limit theorem.

Figure 4 gives the statistical models of constant RCS or RCS fluctuations when the speckle component satisfy the central limit theorem. As Figure 4 shows, many classical statistical models, called the Gaussian model family, have been derived based on the speckle model, a special example of the product model. Either in the high-resolution or low-resolution case, with the hypothesis of a constant RCS background and the central limit theorem, both the I and Q components of the speckle are Gaussian distributed with unit mean. Thus, as is shown in Figure 4, the single-look amplitude has a Rayleigh [1] distribution; the single-look intensity has a negative exponential distribution [1] with unit mean; the multi-look amplitude has a square root Gamma distribution; the multi-look intensity has a Gamma (or Nakagami-Gamma) [1,26,28,59] distribution with unit mean, *etc.*

The RCS of a homogeneous region (e.g., the grassland region) in either low-resolution or high-resolution SAR images can be expected to correspond to a constant. Actually, most scenes contain in-homogeneous regions with RCS fluctuations [1,26,51]. According to Jakeman and Pusey’s [60] investigations, when the number of scatterers in a resolution cell becomes a random variable due to fading phenomenon and the population of scatterers to be controlled by a birth-death-migration process, it should have a Poisson distribution [1] and the mean of the Poisson distribution in each resolution cell (*i.e.*, the expected number of scatterers) itself is also a random variable [24,36,61,62]. If the mean is Gamma distributed, the corresponding intensity data should have a *K* [1,30,60,63–67] distribution. A further research indicates that *K* distribution can be viewed as the combination of two split parts according to Equation (2) in the framework of the product model [1]: (1) the speckle component satisfying the central limit theorem; (2) the Gamma distributed intensity RCS fluctuations. The *K* distribution is deduced with the assumption that the underlying intensity RCS fluctuations have a Gamma distribution in a heterogeneous region. The Gamma distribution can well describe the characteristics of the RCS fluctuations of a heterogeneous terrain in high-resolution SAR images. The deduced *K* distribution itself has the multiplicative fading statistical characteristics and usually provides a good fit to the heterogeneous terrain. Therefore, the *K* distribution has become one of the most widely used and the most famous statistical models in recent years [60,68,69]. Some extensive applications of the *K* distribution can be found [36]. Oliver proposed a correlated *K* distribution [61]; Jao used a *K* distribution in the case of rural illuminated area [68]; Barakat obtained the *K* distribution in case of weak scattering [70]; and Yueh created and extension of the *K* distribution for multipolarization images [62]. Furthermore, according to the deducing process of the *K* distribution, the homogeneous region with a constant RCS can also be described as a special case of the *K* distribution [1]. MoM turns out to be feasible for the parameter estimation task concerning a *K*-distributed random variable [64,65], whereas no closed form is currently available for ML parameter estimation [30,65], thus requiring intensive numerical computations or analytical approximations of the PDF itself [1,26].

**Figure 5.**
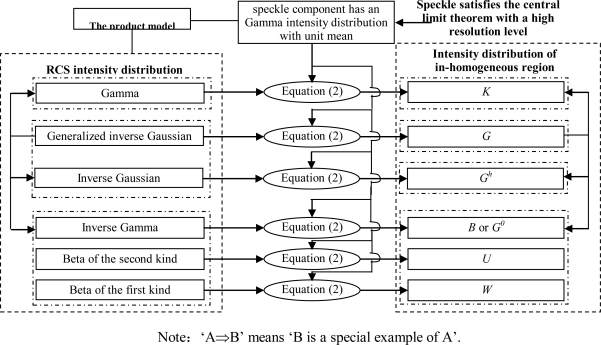
Statistical models of RCS fluctuations when the speckle component satisfies the central limit theorem.

Motivated by the derivation of *K* distribution, Delignon [36,71] proposed that when the expected number of scatterers in every resolution cell has an inverse Gamma intensity distribution [36,71], a Beta intensity distribution of the first kind [36,63,71] or a Beta intensity distribution of the second kind [36,63,71], the corresponding heterogeneous region will has a *B*, *U* or *W* distribution respectively (*i.e.*, the Pearson system of parametric families [17,71]). Similarly, these three kinds of intensity distribution models can be seen as the combination of the speckle component and the terrain RCS intensity component in the framework of the product model expressed as Equation (2). Figures 4 and 5 show the statistical models when the speckle component satisfies the central limit theorem.

The *K*, *U* and *W* distributions have been reported to be appropriate for the heterogeneous terrain such as the woodland and the cultivated cropland. But they cannot meet the demand for the statistical modeling of complex scenes in high-resolution images. The complexity of the high-resolution scenes mainly lies in two aspects [51]: (1) the terrain of the scene is usually extremely heterogeneous, such as the urban region containing many buildings, which results in the severe long-tailed part of the image histogram; (2) there exist two or more heterogeneous components in a certain scene, such as a combination of woodlands and grasslands, *etc.*

To solve these problems, Frery deduced a new statistical model, the *G* model [19,24,72–75] based on the product model assuming a Gamma distribution for the speckle component of multi-look SAR images and a generalized inverse Gaussian (GIG) law for the signal component [24,26,74,76], as is shown in Figure 5. It was Frery who first proposed to divide a region as homogeneous, commonly heterogeneous or extremely heterogeneous according to its homogeneous degree when deducing the *G* model. The *K* and *G*^0^ (also called *B* distribution) distributions are two special forms of the *G* model.

The former is appropriate for the heterogeneous region and the latter is appropriate for the extremely heterogeneous region. The *G*^0^ distribution can be converted into the *β′* (Beta-Prime) distribution under the single-look condition. Although the *G*^0^ distribution is a specific example of the *G* model, it has a more compact form in comparison with the *G* model and consequently has a simple parameter estimation method. The relationship between the *G*^0^ distribution and the *K* distribution cannot be deduced theoretically. The parameters of the *G*^0^ distribution are sensitive to the homogeneous degree of a region, which makes the *G*^0^ model appropriate for modeling either heterogeneous or extremely heterogeneous region. Moreover, MoM can be easily and successfully applied to parameter estimation of the *G*^0^ distribution. Frery [24,72] and Muller [73,74] carried out experiments on many SAR images of different kinds of terrain with various band, polarization, resolution and look numbers, such as different urban areas, homogeneous and heterogeneous regions, *etc.* Their results testified the good characteristics of the *G*^0^ distribution.

A further particular case of the the *G* model (named the “harmonic brach” *G^h^* assuming that the intensity RCS fluctuations of the background are the inverse Gaussian (IG) distribution which has also been employed to model the intensity statistics [24,74]) is proposed in [74] and endowed with a moment-based estimation approach to images of urban areas and mixed terrain.

Eltoft [77–79] assumed a normal IG distribution for the real and imaginary parts of the backscattered complex signal, thus resulting in an amplitude PDF (*i.e.*, “Rician inverse Gaussian”, RiIG) formulated as a combination of an IG PDF and a Rice PDF (see Section 4.4). The purpose of their investigation is to describe the statistics of ultrasound images. While given the similarities between SAR and ultrasound, RiIG can also be used as a model for SAR images. Finite applications of statistical modeling for SAR images can be found in [79]. Anyway, further experimental investigation using real SAR data is needed.

**Figure 6.**
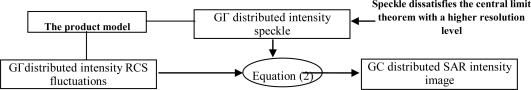
Statistical models when the speckle component dissatisfies the central limit theorem.

The above models developed from the product model are all derived under the hypothesis that the speckle component satisfies the central limit theorem. Theoretically, when the resolution becomes high enough, the resolution cell will be so small that the central limit theorem cannot be applied any more. Thus, the above models are not appropriate for modeling of the high-resolution SAR images. Accordingly, Anastassopoulos [33,80–82] proposed a generalized compound probability distribution (GC distribution, see Figure 6) in which the speckle and intensity RCS fluctuation components theoretically are generalized Gamma distributed (GΓ distribution) [33]. The GC distribution has no analytic expression only with a given integral form, so it is difficult to utilize. With a large number of experiments, we [38] have proven that even if the resolution is high up to 0.3 m, the speckle component still satisfy the central limit theorem. So it is not necessary to adopt the GC distribution for SAR images with a resolution lower than 0.3 m. Besides, due to the absence of the higher-resolution data, further experiments are needed for validating the rationality of the GC distribution.

### 4.2. The Statistical Model Developed from the Generalized Central Limit Theorem

Another thread of statistical modeling is to develop the models based on the generalized central limit theorem [51]. According to the knowledge of probability theory, the generalized central limit theorem states that the sum of a set of independently identically distributed random variables, no matter their variances are finite or infinite, will converge to the α-stable distribution [2,83–85], which is essentially a more general distribution model. Tsakalides *et al.* [83] and Pierce [84] therefore considered that the symmetric α-stable distribution (SαS) [86,87] should be applied to model the real and imaginary parts of the data separately received by the SAR system. The empirical fitting results obtained by Kappor [85] and Banerjee [88] indicated that the SαS distribution could describe some woodland regions in the UWB-SAR images.

In order to consider further the statistical modeling problem of narrowband SAR images, Kuruoglu [3,51,89] introduced the generalized heavy-tailed Rayleigh amplitude distribution based on the SαS (here after simply denoted by SαSGR), which can fit the urban SAR images with a long tail. It can be proved that this distribution is a compound Rayleigh distribution [89,90] and a spherical invariant random process (SIRP) [91]. The SαSGR is a more accurate statistical model of SAR images in theory, without any analytic expression. A moment-based estimation strategy is developed in [51] for this parametric model. However, it is very difficult to apply.

### 4.3. The Empirical Distributions

The empirical distributions have no sound deduction in theory. They come from the experience of analyzing real data. Several empirical models have been used to characterize the statistics of SAR amplitude or intensity data, such as Weibull, log-normal, and Fisher PDFs.

The log-normal distribution was proposed by George [92]. Its major motivation was to adopt a homomorphic filter to convert the multiplicative noise in a SAR image to the additive Gaussian white noise with the assumption that the logarithmic SAR image was Gaussian distributed. The log-normal distribution, with a broad dynamic range, is a familiar statistical model which can describe the non-Rayleigh data. But it is a poor representation of the lower part of the SAR image histogram, with the data over-fitted phenomenon [51,93]. Fukunaga [94] stated that it was inappropriate to fit the logarithmic SAR image to a Gaussian distribution, and that the quarter power domain of the logarithmic data was more consistent with a Gaussian distribution.

The Weibull distribution [95] is also a good statistical model of the non-Rayleigh data. Compared with the log-normal distribution, it can fit the experimental data in a broader range. The Rayleigh distribution and the negative exponential distribution are two special examples of Weibull distribution with specific parameters. Therefore, Weibull distribution can describe single-look images precisely for either amplitude or intensity. Experiences have shown the Weibull distribution cannot represent multi-look images exactly [1].

Recently, the Fisher distribution has also been adopted as an empirical model for the SAR statistics over high resolution urban regions [17,96]. The Fisher distribution also is proved to be equivalent to a *G*^0^ PDF [17,26].

### 4.4. Other Models

Goodman [17,26,59,97] has presented that when a resolution cell is dominated by a single scatterer, the corresponding intensity image has a Rician distribution (or Nakagami-Rice distribution [1]). Theoretically, in the case of low resolution, when the strong scatterers representing the targets are embedded into the surrounding weak clutter environment, the Rician model is appropriate to describe the corresponding image [59,98].

Blake [37,99] introduced a joint distribution model when considering two or more than two heterogeneous terrain types in the scene of a SAR image. Firstly, the optimal statistical model of a homogeneous region is analyzed and the *K* distribution is proven as the best model by the experiments. Secondly, according to the ratio of each terrain to the whole scene, several *K* distributions weighted with the ratios respectively are summed up to describe the image. The unknown parameters of the joint distribution model increase several times in number and thus makes the parameter estimation more difficult. Generally, such parameter estimation is based on solving a set of nonlinear equations [32,64,100], which will impede the application of the joint distribution.

Blacknell [101,102] proposed a statistical distribution model considering the correlation between pixels. Since the pixels of a real SAR image are usually dependent, there is certain correlation between the pixels. Blacknell adopted the mixed Gaussian distribution to model the correlation between the pixels and deduced a statistical model. In fact, the mixed Gaussian distribution can describe only the simplest case of the correlation between the pixels. Further researches are expected for more complicated cases [61,101,102].

Besides, some other models, which are mostly the generalization or modification of the models mentioned above, have been proposed in the literature [103–105], but given the length limitations of this review, they are not not discussed further.

## 5. The Relationship among the Major Models and Their Applications

### 5.1. The Relationship among The Parametric Statistical Models

The statistical model of a single-look image is a special example of the corresponding multi-look model when the look number *n*= 1. Let *P_I_*(*I*) be the PDF of the intensity *I* and *P_A_*(*A*) be the PDF of the amplitude, then the following relationship holds [1]:

(3) $$P_{A}\;(A)=2A\cdot P_{I}\;(A^{2})$$

or:

(4) $$P_{I}\;(I)=P_{A}\;\left(\sqrt{I}\right)/2\sqrt{I}$$

**Figure 7.**
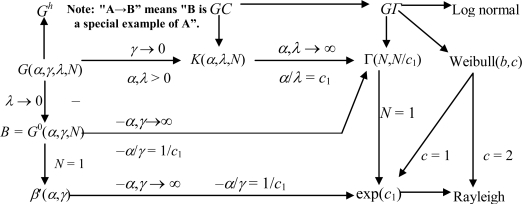
Relationship among the major statistical models (*N* is the look number).

Hence, the statistical distribution of single-look data can be deduced from that of multi-look data; and the distribution of the amplitude can be deduced from that of the intensity. Additionally, the log-transformed distributions are also deduced easily according to [57]. Based on this conclusion, Figure 7 illustrates the relationship among the current major statistical models. Some other models are not shown in Figure 7 because no theoretical relationship for them can be established to the models in Figure 7. The concrete expressions of various distributions can be seen in [2,19].

### 5.2. Summary of the Applications of the Major Models

According to many researchers’ experiences [1] and the authors’ analysis, Table 1 summarizes the characteristics and the application areas of the major models discussed in the previous sections.

## 6. Discussion of Future Work

Much progress has been made with the research of statistical modeling of SAR images in the past few tens of years, especially during recent years. The related literatures are uncountable. As far as we could comprehend, the major conclusions and several promising directions for further research are summarized as follows:

1. Regarding the deducing process of current statistical models, many assumptions are made to acquire the models, so these models can only approximately describe the electromagnetic scattering characteristics of the scene in theory, which is the common shortcoming of all the statistical modeling of the scene. How to construct models that can exactly describe the electromagnetic scattering characteristics of a scene will be a big challenge.
2. Among the existing statistical models, those developed from the product model are the most widely used and the most promising. This can also be seen from the related literatures.
3. The statistical models based on the product model can be divided into two cases according to whether the speckle component satisfies the central limit theorem or not. Correspondingly, there are two typical models, *i.e.*, the widely used *G*^0^ model and the GC model with difficulty in application. The problem is, what level on earth the resolution is increased to that the speckle component doesn’t satisfy the central limit theorem any longer. No conclusion has been made yet.
4. It is a novel idea to model a region according to its homogeneousness degree. The *G*^0^ model (the *β′* model at single-look case) is the optimal one among the models developed from the product model. On one hand, the parameters of the *G*^0^ model are sensitive to the homogeneousness degree of the observed images. Such a characteristic make it suitable for modeling the homogeneous, heterogeneous or extremely heterogeneous, single-look or multi-look, intensity or amplitude data. That means it can be universally used. On the other hand, many widely used models can be unified to the *G*^0^ model (see Figure 7).
5. All the statistical models, even the *G*^0^ model, can describe the regions only with relatively simple contents and a few terrain types. In other words, the statistical model has the so-called “regional” characteristic. For the large- scale scene, whose contents are complex and terrain types are extremely numerous, it is impractical to use the statistical models with a few parameters to describe the whole image. However, models with too many parameters also cause difficulties in applications. Therefore, it is a trend to build a statistical model with the “regional” characteristic. Typically, Billingsley [35] assess the fit of Rayleigh, Weibull, log-normal, and *K*-distributions to pixel magnitudes in clutter data and show via the *K-S* test that none fit well over the entire range of magnitudes.
6. According to the related literatures, once a model was proposed, it would be applied to diverse images with several bands and different view angles. Usually, their results were good. Generally speaking, the diversity of the band and the view angle of a sensor within a certain scope have slight influence on statistical modeling of the SAR data.
7. It is also a new idea to consider the correlation among the SAR data. In theory, it can expose the statistical characteristics of SAR images more accurately. However, it’s hard to exactly model the correlation. Borghys [100] analyzed the effect on the statistical model caused by the correlation among pixels. His conclusion was that through appropriate down sampling, such effect could be ignored when modeling SAR images.

## 7. Conclusions

Statistical modeling of SAR images is one of the basic research topics of SAR image interpretation. It is of great significance both in theory and in applications. Based on an extensive investigation on the related literatures, this paper begins with the history and current research state of statistical modeling of SAR images. Then, statistical modeling techniques are thoroughly reviewed using the product model as a thread and some major problems are briefly illustrated in order to attract more attentions in this field. We believe that the research will progress widely and deeply due to the demands of SAR image interpretation.

## Figures and Tables

**Table 1.** Summary of the applications of the major models.

**Model families**	**Model**	**Analytic expression?**	**Parameter estimation**	**Application cases**	**Notes**
*1*	*Weibull*	Yes	Complex	High-resolution, amplitude or intensity, single-look	unsuitable for multi-look images
	*Lognormal*	Yes	Simple	Moderately high-resolution, amplitude	Data over fitted phenomenon
	*Fisher*	Yes	Simple	Homogenous, heterogeneous or extremely heterogeneous region, multi- or single-look, intensity or amplitude	Be equivalent to a *G^0^* distribution
*2*	*Rayleigh*	Yes	Simple	Homogenous region, single-look, amplitude	Widely used in interpretation algorithms
	*Exp*	Yes	Simple	Homogenous region, single-look, intensity	Widely used in interpretation algorithms
	*Gamma*	Yes	Simple	Homogenous region, multi-look, intensity	The amplitude distribution corresponding to the square root Gamma.
	*K*	Yes	Complex	Moderately heterogeneous region, multi- or single-look, intensity or amplitude (having corresponding expressions for each case)	Widely used in interpretation algorithms
	*U*, *W*	Yes	Complex	Moderately heterogeneous region, multi- or single-look, intensity or amplitude (having corresponding expressions for each case)	Seldom used in interpretation algorithms
	*G*	Yes	Complex	Homogenous, heterogeneous or extremely heterogeneous region, multi- or single-look, intensity or amplitude (having corresponding expressions for each case)	Difficult to apply
	*G^0^*	Yes	Simple	Homogenous, heterogeneous or extremely heterogeneous region, multi- or single-look, intensity or amplitude (having corresponding expressions for each case)	A special example of the G distribution, also called the *B* distribution, widely used
	*β′*	Yes	Simple	Homogenous, heterogeneous or extremely heterogeneous region, single-look, intensity	A special example of the G^0^ distribution, widely used
	*G^h^*	Yes	Simple	extremely heterogeneous urban areas and mixed terrian	A special example of the G distribution
	*RiIG*	Yes	Simple	Ultrasound images	Further investigation for SAR images is needed
	*GC*	No	Complex	Various image data with an extremely high resolution level	A general form of many other models, difficult to apply, further validation is needed
*3*	*SαS*	No	Complex	Real and imaginary components of SAR data	Used in modeling the woodland regions in UWB SAR data
	*SαSGR*	No	Complex	Long-tailed amplitude image of urban area	Difficult to apply
*4*	*Rician*	Yes	Complex	Low-resolution image with targets in weak clutter	Seldom used
	*jointly distribution*	Yes	complex	Heterogeneous	Difficult to apply
	*mixed Gaussian*	Yes	simple	Considering the correlation between pixels	Correlation is simple, further research is needed

Note: “1” represents the empirical distributions; “2” represents the models developed from the product model; “3” represents the models developed from the general central limit theorem; “4” represents other models.
